# Retraction: Unlocking SME growth: Analyzing the government subsidies’ impact on financing in China

**DOI:** 10.1371/journal.pone.0341378

**Published:** 2026-01-26

**Authors:** The PLOS One Editors

Following the publication of this article [1], the corresponding authors contacted PLOS to request the retraction of the article. The corresponding authors stated that a re-examination revealed errors in the article’s data collection and analysis, including missing data that prevents reproduction of robustness checks and heterogeneity analyses.

The *PLOS One* Editors reviewed the data in consultation with a statistical reviewer and identified the following concerns:

- There appear to be inconsistencies in the descriptive statistics in Table 2. Specifically, for Profitability, the p50 median value is lower than the reported minimum value in the Low-Intensity Government Subsidy Policy Group. The corresponding author YL acknowledged the issue and stated that this potentially arose from the use of incomplete or inconsistent data records, noting that in view of the unavailable data, the authors are unable to rectify logical inconsistencies in the published data.
- The correspondence between the data columns in the supporting information files (Excel column header/title) and the variables listed in Table 2 is unclear and there is insufficient reporting of the methodology used to construct these variables. The corresponding author YL stated that a core variable for conditional subsidies (Cond) is not included in the datasets and that the code used to create it is unavailable.

The corresponding author YL stated that because of these issues all subsequent analyses, including robustness checks (GPS and IV), cannot be validated.

The *PLOS One* Editors consider that the above issues collectively raise concerns about the reliability and validity of the results on which the conclusions of this article [1] are based.

In light of these concerns, and in accordance with the request of the corresponding authors, the *PLOS One* Editors retract this article.

YL agreed with the retraction and apologizes for the issues with the published article. YH did not respond to the final editorial decision. WS and ZW either did not respond directly or could not be reached.
